# Folate (vitamin B9) content analysis in bread wheat (*Triticum aestivum* L.)

**DOI:** 10.3389/fnut.2022.933358

**Published:** 2022-10-21

**Authors:** Jun Zheng, Xingsu Wang, Bangbang Wu, Ling Qiao, Jiajia Zhao, Mohammad Pourkheirandish, Juanling Wang, Xingwei Zheng

**Affiliations:** ^1^Institute of Wheat Research, Shanxi Agricultural University, Linfen, China; ^2^Faculty of Veterinary and Agricultural Sciences, University of Melbourne, Parkville, VIC, Australia

**Keywords:** wheat, folates, genetic variation, accession types, agronomic traits

## Abstract

Vitamin B9, particularly folic acid, is an essential molecule for human health. Wheat flour is one of the major sources of calorie intake by humans. The selection of folate-rich genotypes in wheat breeding can enhance the natural folate value in the daily diet. This study used a precise, high-performance liquid chromatography (HPLC) assay to analyze folate content in a 262-accession Chinese wheat mini-core collection (MCC) grown under three environments. Four folate derivatives in grains including tetrahydrofolate (THF), 5-methyltetrahydrofolate (5-CH_3_-THF), 5-formyltetrahydrofolate (5-CHO-THF), and 5,10-methenyltetrahydrofolate (5,10-CH^+^THF) were considered. An association analysis of water regimes, accession types, released years, geographical origin, and agronomic traits with folate content was conducted for the first time. There was a large amount of variation in folate content in the analyzed accessions, with genotype identified as the main influencing factor. Total folate content was significantly correlated with the content of the four MCC derivatives under the three environments. 5-CH_3_-THF and 5-CHO-THF were the most abundant among the four folate derivatives and were positively correlated with high folate content. The 12 accessions with the highest folate content showed an average of more than 80 μg/100 g. The analysis demonstrated that this Chinese wheat had not undergone extensive selection for folate content during breeding, which is unrelated to the geographical origin, accession types, winter/spring types, and grain colors of wheat. The content of THF, 5-CH_3_-THF, and 5,10-CH^+^THF was significantly negatively correlated with grain width, grain thickness, and thousand kernel weight. A relatively weak negative relationship manifested between folate contents and flowering date, whereas no significant correlation with tiller number, grain number per spike, maturity date, height, and spike length was detected. The investigation benefits wheat breeders for folate enhancement.

## Introduction

Folate (vitamin B9) represents a class of water-soluble vitamers that can only be synthesized *de novo* by plants and microorganisms (1). The folate vitamers play essential roles in one-carbon metabolism for involvement in the biosynthetic pathways for key amino acids and nucleic acids (2). As such, folate is a key factor in cell replication and intermediary metabolism (3). Insufficient folate intake in pregnant women may lead to low weight, heart disease, and neural tube defects in infants (4). Every year 7.9 million infants suffer from congenital disabilities, and the incidence of neural tube defects accounts for about 10% of them (5, 6). Folate deficiency can increase the risks of Alzheimer’s disease, coronary heart disease, stroke, and certain cancers (7, 8). Animals and humans are incapable of folate biosynthesis and depend on dietary intake of folates (9). The recommended dietary allowance (RDA) of folates by the World Health Organization (WHO) is 200 μg per day for normal adults and 400 μg per day for pregnant and lactating women (10, 11). Inadequate folate uptake is common in poor regions and developing countries. Research on biofortified foods with the possibility of enhancing dietary folate levels has increased in recent years (1, 12, 13).

Folic acid supplementation *via* pills and processed foods and natural folate intake through varied dietary sources are common approaches to combat folate deficiency. However, excessive uptake of synthetic folic acid was reported to cause changes in DNA methylation levels, which has been linked with the risk of colorectal and prostatic cancers in men, as well as cognitive impairment in people with vitamin B12 deficiency (14, 15). Subject to economic constraints, dietary habits, or lack of stable social policy and sustained financial support, many people in the world cannot consume varied diets beyond staple foods (16). Thus, folate-enriched staple crops are a feasible, cost-effective strategy to fight this deficiency worldwide. Wheat is one of the most widely grown and important food crops globally, and whole-grain flours are recommended by dietary guidelines due to the high nutrient content in the seed coat and the germ (17). Folate-rich wheat varieties can be directly used to achieve folate supplementation through a daily diet that would yield human health benefits, especially in poor regions (18). One of the most promising paths forward is to exploit the natural variations of the vitamin in wheat germplasm and study the effects of genetic and environmental factors on folate levels. This knowledge can be utilized in wheat breeding programs to enhance folate contents.

Total folate content in wheat grains was determined using two methods, namely, microbiological assay (MA) and high-performance liquid chromatography (HPLC) (19, 20). MA is an easy and inexpensive way to determine total folate, whereas HPLC is a more precise method that has been used to calculate folate content but is time-consuming and costly (21). Studies in the 1980s found folate dry matter (dm) in American and Canadian bread ranged from 16 to 81 μg/100 g (22). A study of 12 Australian wheat varieties found a much higher total folate range at 65–114 μg/100 g dry weight (23). The highest folate content reported by Piironen et al. (24) was at 97.5 μg/100 g of dm with a threefold difference variation in the total grain folate. A recent study found a 2.8-fold difference (up to 88.9 μg/100 g of dm) in total folate content between 26 wheat genotypes using MA and HPLC assays and suggested that environmental factors more strongly affect folate content than do genetic factors (25). These results collectively demonstrate the potential genetic variation of folate content between genotypes. Extensive screening of wheat germplasms for folates would bring out folate-rich wheat varieties.

In addition to selecting folate-rich varieties, association analysis of folate contents with cultivation conditions and agronomic parameters is vital for enhancing folate levels in wheat-based foods. Multiple studies have shown that environmental factors strongly affect wheat folate content (24, 25). A reliable evaluation of the effects of different irrigation and agronomic traits on folate contents has not been fully achieved. For instance, Piironen et al. (24) reported that low thousand kernel weight was associated with high folate contents in winter wheat, but not in spring wheat. The folate content of 360 wheat samples using HPLC coupled with mass spectrometry (HPLC-MS) has been reported (21). Most wheat genotypes tested in that study were modern varieties released in the last 15 years, and only about ten accessions were examined at different growing locations. Until now, limited data have been available on the influence of environmental factors and agronomic traits on folate contents influenced by environmental factors and agronomic traits.

In this study, an HPLC assay was used to analyze the folate contents of a 262-accession Chinese wheat mini-core collection (MCC) grown under the three environments. The objectives of this study were to (i) investigate the natural variation of folate content in a Chinese wheat MCC; (ii) identify wheat accessions with high folate content; (iii) assess the effects of water regimes, accession types, released years, and geographical distribution on folate level; and (iv) evaluate the association of folate with crucial agronomic traits. The findings would contribute to an increased understanding of folate in wheat, and the elite cultivars identified could be incorporated for folate enrichment diet and breeding.

## Materials and methods

### Plant materials

A Chinese wheat MCC was employed to evaluate the effects of environment and genotype on folate content. The MCC contained 262 wheat accessions, including 157 landraces, 88 modern cultivars, and 17 introduced accessions. The Chinese wheat MCC was screened from 23,090 common wheat accessions representing 1% of the Chinese wheat lines with more than 70% of the genetic diversity (26, 27). The higher genetic diversity and artificial diminishment of dominant allelic frequencies in the MCC make it a suitable sample population for the detection of functional substance and can serve as a reference set for revealing broad-sense heritability, geographical origin, and time changes of functional substance. A local variety Lin 6308, developed by Shanxi Agricultural University, was used as standardization control during the HPLC process.

### Field environment design

The MCC accessions were planted in 2018 under the three environments, viz. well-watered and rain-fed fields at Linfen in Shanxi province (36°2′ W and 111°18′ E) and at Xinxiang in Henan province under the well-watered condition (35°18′ N, 113°54′ E). The well-watered treatment group was irrigated three times with 700 m^3^⋅ha^–1^: before winter and at the jointing and filling stages, while the rain-fed group had no irrigation. The average rainfall in Linfen was 220 mm during the growing season (Supplementary Figure 1).

Each accession was planted in a plot with a 2-m length by 1-m width. Distance between plants within a row was set to 5 cm with 25 cm between rows. A randomized block design with three replications was conducted. Spring wheat genotypes were covered by a plastic film in order to survive the winter. Field management was in accordance with local practice, and no pesticides were used. Normal mature grains were harvested for the grain characteristics tests, as well as the folate content evaluation. The control variety Lin 6080 was planted and harvested same with the MCC accessions.

### Phenotypic traits collection

Agronomic traits of all MCC accessions, including heading date (HD), maturity date (MD), spike length (SL), spikelet number per spike (SN), plant height (PH), grain number per spike (GN), effective tiller number (ETN), grain width (GW), grain length (GL), grain thickness (GT), grain color (GC), and thousand kernel weight (TKW), were collected from 10 plants in the middle of each plot and used for the phenotyping of all characteristics.

### Chemicals and reagents

The folate standards 5-methyltetrahydrofolate (5-CH_3_-THF), 5,10-methenyltetrahydrofolate (5,10-CH^+^THF), 5-formyltetrahydrofolate (5-CHO-THF), and tetrahydrofolate (THF) used as an internal control were purchased from Schircks Laboratories (Jona, Switzerland). Ascorbic acid, β-mercaptoethanol (biotechnology grade), potassium phosphate monobasic (analytical reagent), potassium phosphate dibasic anhydrous (analytical reagent), methanol (HPLC grade), acetonitrile (HPLC grade), and formic acid (HPLC grade) were sourced from Shanghai Macklin Biochemical Co., Shanghai, China. Mouse serum was obtained from Abbkine Scientific Co., Wuhan, China. The stock solution of folate standards was dissolved in solution (1% ascorbic acid, 0.1% β-mercaptoethanol, 20 mM phosphoric acid; pH 7.0) at a concentration of 0.5 mg per mL, stored in a refrigerator at −80°C, and then diluted with distilled water to 10, 20, 40, 80, 160, and 200 ng per mL for use.

### Folate extraction and deglutamylation

Samples were prepared using extraction and dual-enzyme treatment as reported previously (21) with a minor modification. The experimental procedure was carried out under dark conditions. The wheat grains were kept in an electro-thermal constant temperature incubator at 37°C for 72 h. The moisture content of grain samples was determined by AACC 44-15A method and maintained at approximately 12.50–13.00%. For extraction, 100 whole dried grains of each wheat variety were separately ground into a fine powder using a grinder with a 100-mesh sieve (Geno/Grinder 2010). Then, 100 mg of powder was transferred to a 1.5-mL frozen tube, and 1 mL of freshly prepared extraction solution (20 mM phosphate buffer, pH 7.0, 1.0% sodium ascorbate, and 0.2% β-mercaptoethanol) was added. After homogenization, the mixture was immediately boiled for 5 min in a water bath, followed by cooling on ice for 10 min. Then, a 50-μL α-amylase solution (20 mg per mL) was added, and the mixture was incubated at 37°C for 30 min to fully degrade the starch into macromolecules and disaccharides. The mixture was treated in a boiling water bath for 3 min, then cooled on ice for 5 min, followed by the addition of 35 μL of rat serum, and incubated at 37°C for 4 h to deconjugate the polyglutamylated tails. Next, the samples were boiled for 3 min, cooled on ice for 10 min, and then centrifuged at 13,000 rpm at 4°C for 10 min. The supernatants were transferred to 3 kDa ultra-filtration tubes (Millipore, Billerica, MA, USA) for cleanup and centrifuged at 13,000 rpm at 4°C for 30 min. Finally, the resulting filtrate was collected, and 100 μL was transferred to new tubes for direct folate detection, and the remaining solution was stored at −80°C. In addition, an endogenous folate blank was prepared using 100-mg quartz sand instead of wheat samples by following the same procedures of the wheat sample preparation. The final filtrate was confirmed below detection limits. Three biological replications from independent plants were used for each sample.

### Folate determination by high-performance liquid chromatography

Chromatographic analyses were performed on an Agilent 1260 HPLC system (Palo Alto, CA, USA) using an Akzo Nobel analytical column (Kromasil100-5 C18, 2.1 mm × 50 mm) and a Kromasil SB-C18 pre-column (2.1 mm × 5 mm, 2.7 μM particle size) (CA, USA), at a flow rate of 0.30 mL per min. The injected sample was 20.0 μL. The temperature of the injector and column oven was separately maintained at 4 and 25°C, respectively. The detection was carried out using a UV detector set at 280 nm. The mobile phases were 0.1% (v/v) formic acid in water (phase A) and 0.1% (v/v) formic acid in acetonitrile (phase B). The gradient program was run for a total of 8 min and 12 s. The proportion of mobile phase B increased linearly from 5 to 9% over 2 min. In the following 6 min, the proportion of phase B increased to 9.6% and then decreased to 5% in 12 s, followed by a subsequent equilibration. Lin 6308 was used as a control of standardization and was repeated once with every 20 samples. The process was stopped, and a check was made to determine whether the difference in folate content between the two repeats of Lin 6308 exceeded 5%.

The four folate derivative standards were serially diluted and used to set the analysis conditions and determine the retention time. Derivatives were identified by retention time and quantified using a six-point calibration curve regression line generated with the external standards, and LOD and LOQ were defined as the lowest analyte concentration yielding a signal-to-noise (S/N) ratio of >3 and of >10, respectively (2). The linearity of the calibration lines was given. Accuracy was evaluated by the spiking standard with approximately twofold concentrations of the samples, and then, the recovery percentage of the added concentrations of the folate was evaluated.

### Statistical analyses

Total folate contents were given on a dry matter basis, calculated using moisture contents (24). The mean values of phenotypic traits and standard errors were analyzed by SPSS 16.0 software. The mean value of each trait was estimated by the best linear unbiased predictor (BLUP) method (28, 29). One-way analysis of variance (ANOVA, *p* < 0.05) was used to evaluate the effects of water regimes, accession types, released years, spring/winter, grain color, and geographical origin on folate content. *F*-test was used to analyze the homogeneity of error variances. Genetic effects, environmental effects, and their interaction were evaluated by two-way ANOVA using SPSS (*p* < 0.001). The relationship between folate content and agronomic traits was analyzed using correlation analysis. The Pearson correlation coefficients were calculated using Statgraphics Plus 4.0.

## Results and discussion

### Extraction and recovery studies by high-performance liquid chromatography

Previous studies showed that four folate derivatives including THF, 5-CH_3_-THF, 5-CHO-THF, and 5,10-CH^+^THF mainly account for the total folate in wheat grains (19, 21, 30). This study analyzed these four derivatives in the grains of a 262-wheat accession MCC using the HPLC method. The elution order and corresponding retention times were THF (1.686 min), 5-CH_3_-THF (2.302 min), 5-CHO-THF (5.751 min), and 5,10-CH^+^THF (6.496 min) (Supplementary Figure 2). Quantification of folate vitamers was based on a standard external method, with peak areas plotted against concentrations (Supplementary Figures 3A–D) (31). High linearity with an *R*^2^ value of the calibration curve >0.99 was confirmed for the four derivatives (Supplementary Figures 3E–H). The average spiked level recoveries are given in Supplementary Tables 1, 2. High percentage recoveries (95.31–99.06%) and acceptable variations (RSD%<5) were obtained for the four investigated folate derivatives.

### Overall variation of the folate levels in Chinese wheats

**FIGURE 1:**
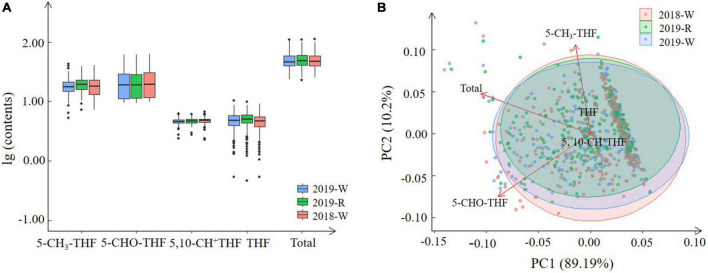
Natural variation **(A)** and score plots **(B)** of total folate and four derivatives levels among the wheat genotypes harvested under the three environments.

In total, 262 wheat varieties were collected from the three environments: 2018 in Xinxiang well-watered (2018-W), 2019 in Linfen well-watered (2019-W), and 2019 in Linfen rain-fed (2019-R) treatments. Significant variations in the total folate levels were observed among the wheat genotypes. The ranges of total folate levels were 23.29–109.58 μg/100 g (2019-W), 22.68–108.75 μg/100 g (2019-R), and 25.41–111.77 μg/100 g (2018-W), and the average contents were 50.52, 51.33, and 50.30 μg/100 g, respectively (Figure 1A and Supplementary Table 4). The accessions were classified based on folate levels: The largest group of accessions (50.78%) were in the range of 35–50 μg/100 g, followed by the group (26.17%) in the range of 50–65 μg/100 g (Supplementary Table 3). Under the three environments, 12 varieties showed an average total folate content higher than 80 μg/100 g. The Wumangchunmai, Youmangbaifu, and Fuyanghong accessions exhibited the highest values, with Wumangchunmai the highest at 110.03 μg/100 g (Supplementary Table 4).

Our folate content results were comparable to those of previous studies. Piironen et al. (24) reported the folate contents of 150 bread wheat genotypes ranging from 32.3 to 77.4 μg/100 g of dm, including Atlas 66, a well-known wheat variety with high folate content (65.0–69.9 μg/100 g), which is consistent with the folate content of 75 μg/100 g observed in this study. However, the highest (77.4 μg/100 g) folate content reported by Piironen et al. (24) was lower than the highest levels in this study. This difference could be explained by genetic differences in the materials, as Piironen et al. (24) used almost exclusively modern varieties, or by differences in growing conditions between the two studies. According to Kariluoto et al. (25), the folate levels of 24 winter wheat and two spring wheat genotypes ranged from 32.3 to 88.9 μg/100 g of dry matter. Recently, Riaz et al. (21) reported total folate content among wheat genotypes in North China ranging from 10.15 to 91.44 μg/100 g, which is consistent with this study. Our results showed that the folate content in the richest variety was nearly fivefold higher than in samples with the lowest folate levels, revealing a large variation in the folate content of this genotyping panel. Moreover, 12 varieties with total folate content above 80 μg/100 g were identified. At those levels, an average consumer would have to eat around 200 g of flour each day to provide the RDA of vitamin B9.

The wheat variety Youmangbaifu showed the highest THF in 2019-W and 2019-R with 10.40 and 9.93 μg/100 g, respectively. Dakoumai showed the highest level in 2018-W with 9.24 μg/100 g. Dunhuachunmai showed the lowest THF in 2019-W, 2019-R, and 2018-W with 0.54, 0.47, and 0.54 μg/100 g, respectively. There were five varieties with an average THF content >8 μg/100 g, including Youmangbaifu (9.68 μg/100 g), Dakoumai (8.68 μg/100 g), Zhugoumai (8.35 μg/100 g), Jiangmai (8.34 μg/100 g), and Huoqiu (8.04 μg/100 g) (Supplementary Table 4).

5-CH_3_-THF is the most active among folate forms (18, 32). The genotypes with the highest 5-CH_3_-THF content under all three environments were Youmangbaifu (40.41 μg/100 g), Wumangchunmai (38.24 μg/100 g), Fuyanghong (34.00 μg/100 g), Zhumaoyuanzitou (33.76 μg/100 g), and Nanda 2419 (30.95 μg/100 g). Among them, Youmangbaifu in 2019-W exhibited the highest level at 42.34 μg/100 g (Supplementary Table 4). The lowest average 5-CH_3_-THF content under the three environments was found in Fengmai 11 (9.29 μg/100 g) and Dabaimai (9.56 μg/100 g).

5-CHO-THF is another abundant folate form in wheat grains (11, 21, 33). Wumangchunmai showed the highest 5-CHO-THF levels under all three environments, with a value of 62.88 μg/100 g in 2018-W. The genotypes with the highest 5-CHO-THF content under all three environments were Wumangchunmai, Dongfanghong 3, Xiaokouhong, Lumai 1, and Qiangchangmai, and there were 14 genotypes overall with an average 5-CHO-THF content >50 μg/100 g (Supplementary Table 4). Both Dahuangpi and Mazhamai showed the lowest 5-CHO-THF values with 9.47 μg/100 g in 2019-W.

Genotypes showing the highest 5,10-CH^+^THF content were Rikaze 8 (6.66 μg/100 g) in 2018-W, Zhahong (6.27 μg/100 g) in 2019-W, and Tuokexun 1 (6.25 μg/100 g) in 2018-W. The genotypes with the highest average contents under the three environments were Zhahong (5.74 μg/100 g), Bendihuanghuamai (5.58 μg/100 g), Zhongyou 9507 (5.52 μg/100 g), Bailanghuimai (5.49 μg/100 g), and Zhuoludongmai (5.42 μg/100 g) (Supplementary Table 4). The genotypes with the lowest average contents under the three environments were Baimai 26 (2.93 μg/100 g) and Fengmai 11 (2.67 μg/100 g).

### Folate form composition in Chinese wheat genotypes

5-CHO-THF and 5-CH_3_-THF make up the majority of folate in wheat grains at about 70% (11, 33, 34). This study shows a distribution pattern of the four derivatives consistent with previous research, with 5-CHO-THF and 5-CH_3_-THF being the predominant folate forms (Supplementary Figure 4).

Folate form distribution studies reported by Piironen et al. (24) and Riaz et al. (21) both found the quantities of 5-CHO-THF and 5-CH_3_-THF to be negatively associated with each other. In this study, the folate form contents were classified into four levels (Table 1). No significant correlation was observed between 5-CHO-THF and 5-CH_3_-THF contents at the lowest level (<15.3 μg/100 g). In the next two levels (15.3–18.9 μg/100 g and 18.9–22.6 μg/100 g), 5-CH_3_-THF has a very weak negative correlation with 5-CHO-THF. However, 5-CH_3_-THF and 5-CHO-THF were positively correlated when 5-CH_3_-THF was higher than 22.6 μg/100 g. These results do not conflict with those of previous reports, and we speculate that the larger number and more diverse accessions used in this study exhibited more variable folate contents, which may provide theoretical support for the breeding of high-content folate varieties.

**TABLE 1:** Correlation analysis between the folate derivatives 5-CH_3_-THF and 5-CHO-THF.

5-CH_3_-THF	*r*
<15.3 μg/100 g	0.289
15.3–18.9 μg/100 g	−0.010
18.9–22.6 μg/100 g	−0.165
>22.6 μg/100 g	0.450*

*r* Indicates the correlation coefficients between 5-CH_3_-THF and 5-CHO-THF.

*Represents the significance level calculated at *P* ≤ 0.05.

### Effects of genotype, environment, and their interactions

Correlation analysis of the three environments was performed using the content values of total folate and four derivatives (THF, 5-CH_3_-THF, 5-CHO-THF, and 5,10-CH^+^THF) of the MCC. The results demonstrated a significant effect of genotype on the content of all four forms of folate (Table 2). However, only 5-CH_3_-THF and 5,10-CH^+^THF showed a significant effect of environmental conditions, suggesting that environmental factors played a weak role in determining folate content and that genotypes might be the key influencing factors.

**TABLE 2:** Effect of genotype, environment, and their interaction on folate content.

Variables	G	E	G × E
THF	58.803**	10.280	30.916**
5-CH_3_-THF	38.095**	32.777**	29.128**
5-CHO-THF	95.304**	0.075	4.621**
5,10-CH^+^THF	28.476**	19.779**	51.745**
Total	83.049**	7.706	9.245**

Sign ** represents the significance level calculated at *P* ≤ 0.001.

The percentage of the total mean square for each variable (G, E, and G × E) was determined to quantify the contribution of each variable to folate levels. The results showed that both G (*p* < 0.001) and the G × E interaction (*p* < 0.001) had highly significant effects on the content of total folate and the four derivatives (Table 2). Only 5-CH_3_-THF and 5,10-CH^+^THF showed a significant effect of environment on variation. G accounted for the most variation in total folate content (83.049%, *p* < 0.001), THF (58.803%, *p* < 0.001), and 5-CHO-THF (95.304%, *p* < 0.001). The largest variation accounted for by E was in the 5-CH_3_-THF level (32.777%, *p* < 0.001), and the lowest variation was in 5-CHO-THF content (0.075%). In general, the effect order on the variation of the total folate content, THF, and 5-CHO-THF was G > G × E > E. The effect order on the variation of 5-CH_3_-THF was G > E > G × E, whereas, the effect order on the variation of 5,10-CH^+^THF was G × E > G > E.

We found genotype had a larger effect on variance than did environment in folate levels in the 262 MCC accessions grown under three different environments. This differs from Riaz et al. (21), who reported that environment and the interaction of genotypes had a large effect on folate production in seven genotypes grown in three different locations. Significant variation in folate levels was also observed in 26 wheat genotypes grown in four different locations (25), and environmental factors affected folate content more strongly than did genetic factors. Compared with this study, the number of wheat accessions analyzed in different regions in previous research is considerably limited. Furthermore, winter/spring types could influence wheat growth in different locations. Wheat is the most widely distributed crop in the world, grown from latitude 18–50° in the North, from plains to plateaus, and up to an altitude of 4,000 m (35). In this research, 2019-W and 2019-R are two environments in the same location differing by water regime. Folate contents in wheat grown in 2019-W and 2019-R were strongly positively correlated, and the genotypes with the highest folate levels between the two environments are basically consistent (Figure 1B). This indicates that water may be less responsible for folate content in wheat and the potential of folate biofortification in wheat is much greater than that of maize and rice, which have higher water requirements. The amount of empirical data could provide more precise information regarding the degree of effects of genotype, environment, and their interactions in folate contents.

### Distribution of folate content in Chinese wheat varieties

**FIGURE 2:**
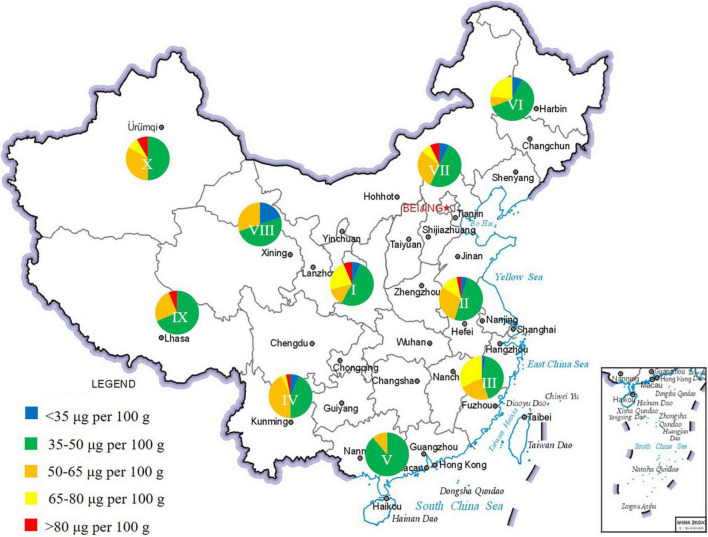
Frequency distribution of folate contents in agro-ecological production zones.

Chinese wheat was divided into 10 major agro-ecological zones based on differences in local climatic conditions, regional types, and planting systems (36). By analyzing the folate contents of varieties from different wheat zones, the influence of geographical origin on folate levels can be interpreted. The proportion of varieties distributed within different ranges of total folate contents in each zone was approximately the same (Figure 2 and Supplementary Tables 5, 6). No significant differences were observed in average folate contents among the 10 zones, except for zone V which mainly constitutes varieties with lower folate content (Figure 2). This result further indicated that folate contents are mainly affected by genotype, suggesting that geographical restrictions should be of less consideration in varieties breeding with high folate levels.

### Association of wheat folate contents with accession type

**FIGURE 3:**
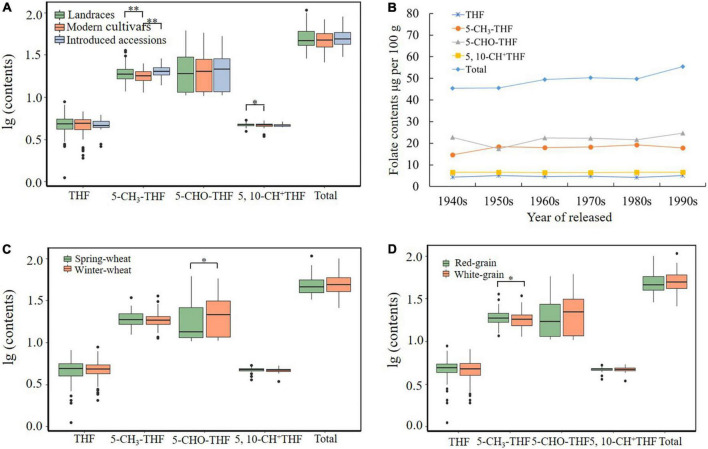
Total folate and derivatives contents affected by accession types **(A)**, released years (landraces not included) **(B)**, winter/spring types **(C)**, and grain colors **(D)**. * and ** indicate significant differences at 0.05 and 0.01 levels, respectively.

The MCC contained lines, including landraces, modern varieties, and introduced lines, that are suitable for evolution analysis of agronomic traits during wheat breeding (27, 37, 38). We analyzed folate distribution among the three types of wheat accessions (Figure 3A). The THF content in landraces and modern varieties has a wider range of variation than that in introduced varieties. THF, 5-CH_3_-THF, 5-CHO-THF, 5,10-CH^+^THF, and total folate content showed no significant variation among genotypes from different types, suggesting that the folate level trait might not have undergone strong selection during wheat breeding. Overall, landraces are expected to have a higher genetic diversity compared with modern varieties, and they may contain more genotypes with high folate content. It is necessary to analyze the genetics of these resources and find genes related to high folate, which could help the design of functional markers to assist in folate-rich breeding.

### Association of wheat folate contents with wheat breeding and selection

The content of total folate and four folate derivatives in Chinese wheat MCC accessions released in different years was compared and analyzed. Over time, the average total folate content remained basically unchanged at the level of 50 μg/100 g, and the content of the four derivatives also showed no significant change, indicating that the folate content trait has not been artificially selected for or against during Chinese wheat breeding (Figure 3B).

### The effect of winter/spring types of common wheat on folate contents

Comparing the content of four folate derivatives and total folate in different growth habit varieties revealed the 5-CHO-THF content of spring wheat to be significantly lower than that of winter wheat (20.22 ± 11.67 μg/100 g vs. 23.97 ± 13.38 μg/100 g), while there was no difference between the seasonal varieties for the other three derivatives or total folate, with the total folate content of 51.84 ± 14.30 μg/100 g in winter wheat and 49.54 ± 13.78 μg/100 g in spring wheat (Supplementary Table 7 and Figure 3C), indicating that the growth habit has little effect on the content of folates. Consistent with our results, Piironen et al. (24) reported that the average total folate content of 20 spring wheat genotypes was 55.1 μg/100 g.

### Effect of grain colors on folate contents

Red and white are the most common wheat grain coat colors, which are often related to the accumulation of the polyphenolic compound phlobaphene in the outer layer of grains (39, 40). Previous reports indicated a red-pigmented rice grain variety accumulated a twofold higher folate concentration than that found in white rice grain (20). There are 167 red-grained and 95 white-grained accessions among the MCC, and the effect of grain coat color on wheat folate content was analyzed (Figure 3D). The results showed that the average content of 5-CH_3_-THF in red-grained wheats (19.30 μg/100 g) was significantly higher than in white-grained wheats (18.35 μg/100 g). The contents of THF, 5-CH_3_-THF, 5,10-CH^+^THF, 5-CHO-THF, and total folate were not affected by grain color (Supplementary Table 7). The folate contents in this study have no significant difference between the two grain color wheats.

### Effect of grain and agronomic-related traits on folate contents

Several studies reported folate contents to be negatively correlated with grain size (21, 24, 25). However, none of these studies analyzed the grain size component with different heritability. In the current study, grain-related traits were divided into three categories, namely, grain length (GL), grain width (GW), and grain thickness (GT), which were correlated with folate contents. The contents of THF, 5-CH_3_-THF, and 5,10-CH^+^THF were significantly negatively correlated with GW, GT, and thousand kernel weight (TKW) (*p* < 0.01), but uncorrelated with GL (Table 3). These data indicate decreasing content of three of the folate forms with the increase in GW, GT, and TKW. The levels of both 5-CHO-THF and total folate were slightly negatively correlated with GL, GT, and TKW. The negative correlation of wheat folate content with kernel size is well in line with previous results (21, 24, 25). Therefore, the genotypes with small grains having low GL, GT, and TKW would be predicted with high folate levels.

**TABLE 3:** Correlation of folate contents and important agronomic traits.

Type	ETN	GN	HD	MD	PH	SL	SN	GL	GW	GT	TKW
THF	0.058	–0.036	−0.202**	0.065	0.078	–0.005	0.047	–0.095	−0.153*	–0.108	−0.198**
5-CH_3_-THF	–0.042	0.058	−0.152*	0.026	–0.042	–0.098	0.087	0.043	−0.219**	−0.167**	−0.204**
5-CHO-THF	–0.011	0.116	−0.102*	0.003	–0.075	–0.106	–0.02	–0.097	0.055	–0.014	–0.014
5,10-CH^+^THF	–0.001	0.019	−0.167**	0.079	0.026	–0.027	0.017	–0.093	−0.158*	−0.147*	−0.199**
Total	–0.015	0.072	−0.145*	0.019	–0.073	–0.085	0.003	–0.089	–0.038	–0.079	–0.101

ETN, effective tiller number; GN, grain number per spike; HD, heading date; MD, maturity date; PH, plant height, SL, spike length; SN, spikelet number per spike; GW, grain width; GL, grain length; GT, grain thickness; GC, grain color; TKW, thousand kernel weight. * and ** represent significant difference at the 0.05 and 0.01 probability levels, respectively.

Correlation analysis between folate contents in Chinese wheat MCC and seven agronomic traits was performed. No significant correlation was detected between folate derivatives and effective tiller number, grain number, maturity date, height, spike length, and spikelet number, indicating that these agronomic traits have no effect on folate levels in wheat (Table 3). A negative relationship between heading date and folate contents was detected, especially in THF content (*r* = −2.02, *p* < 0.01). Even though this correlation is relatively weak, the finding suggested that early flowering will lead to a higher THF level, which may be explained by an accumulation of the folate during the extended filling stage. Accessions with high temperature tolerance and long filling periods should be selected in favor of high folate content. In addition, cultivation practices to extend the filling period may be useful not only for facilitating the accumulation of folate, but also for obtaining higher yields.

## Conclusion

In conclusion, this study provides new data on variation in folate contents of bread wheat. The large number of genotypes and experimental environments provided a unique opportunity to obtain reliable information on folate in wheat breeding. There is a large variation in the folate contents of Chinese wheat accessions, and we identified 12 that can provide excellent resources for high-folate-level wheat breeding. Genotype was the key factor determining folate contents in wheat, and the potential for folate biofortification in wheat is high given that it is a major crop. Folate levels in Chinese wheat have not undergone selection and are not related to the originating ecological regions, accession types, growth habit, or grain colors of wheat. 5-CH_3_-THF and 5-CHO-THF were the main derivatives of folates; they can be improved together in the selection of folate-rich varieties. Folates were observed to negatively relate to wheat flowering date, which provides a basis for breeding and cultivation practices for folate biofortification. Future research should involve a GWAS analysis of the same material to find the genomic region responsible for folate synthesis in wheat.

## Data availability statement

The original contributions presented in this study are included in the article/Supplementary material, further inquiries can be directed to the corresponding authors.

## Author contributions

XZ and JW were involved in conceptualization, methodology, and supervision. MP was involved in writing—review and editing and supervision. JuZ and XW were involved in data curation and writing—original draft preparation. BW was involved in methodology and software. LQ and JiZ were involved in visualization and investigation. All authors contributed to the article and approved the submitted version.
