# Supplementary material for: Folate (vitamin B9) content analysis in bread wheat (Triticum aestivum L.)
Source: Front Nutr. 2022 Oct 21;9:933358. doi: 10.3389/fnut.2022.933358 (PMC9633958; doi:10.3389/fnut.2022.933358)
Supplement: Supplementary file 1 [file Data_Sheet_1.docx]

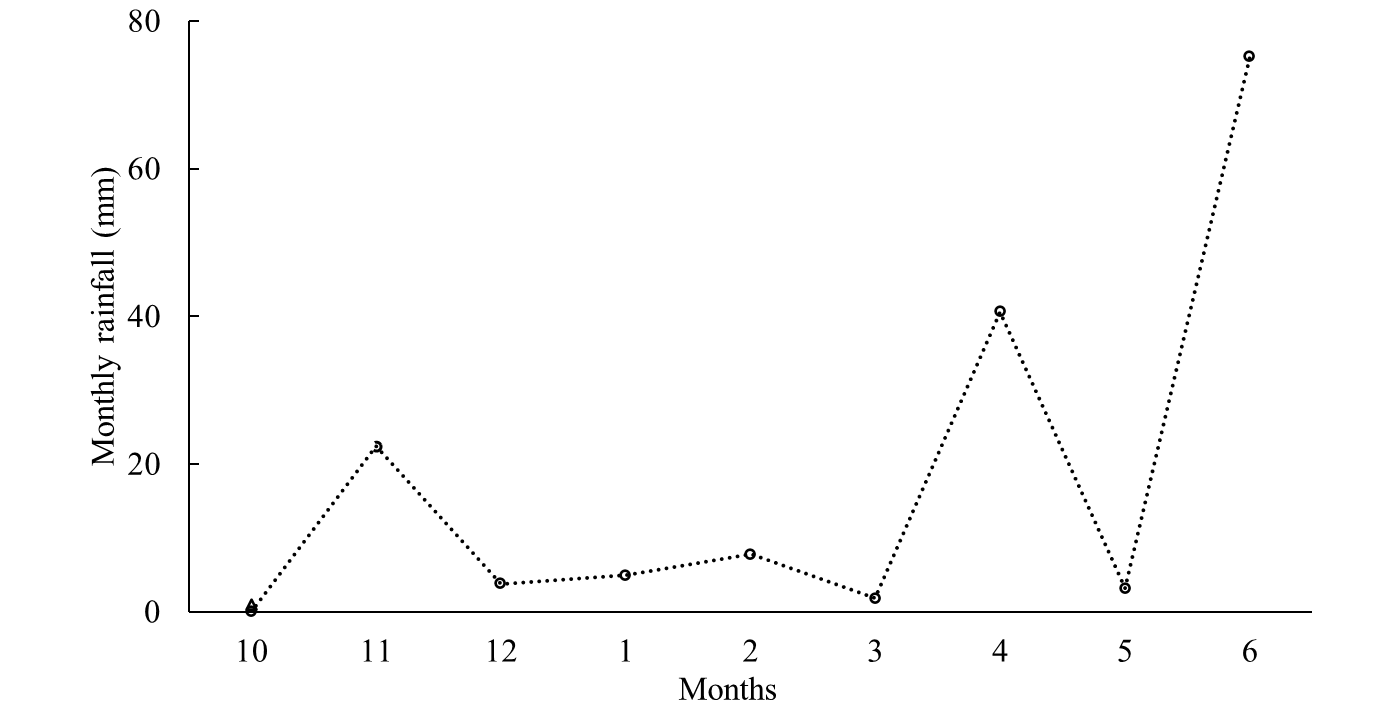


Supplementary Fig.1. Monthly Rainfall of two years during the growing season.


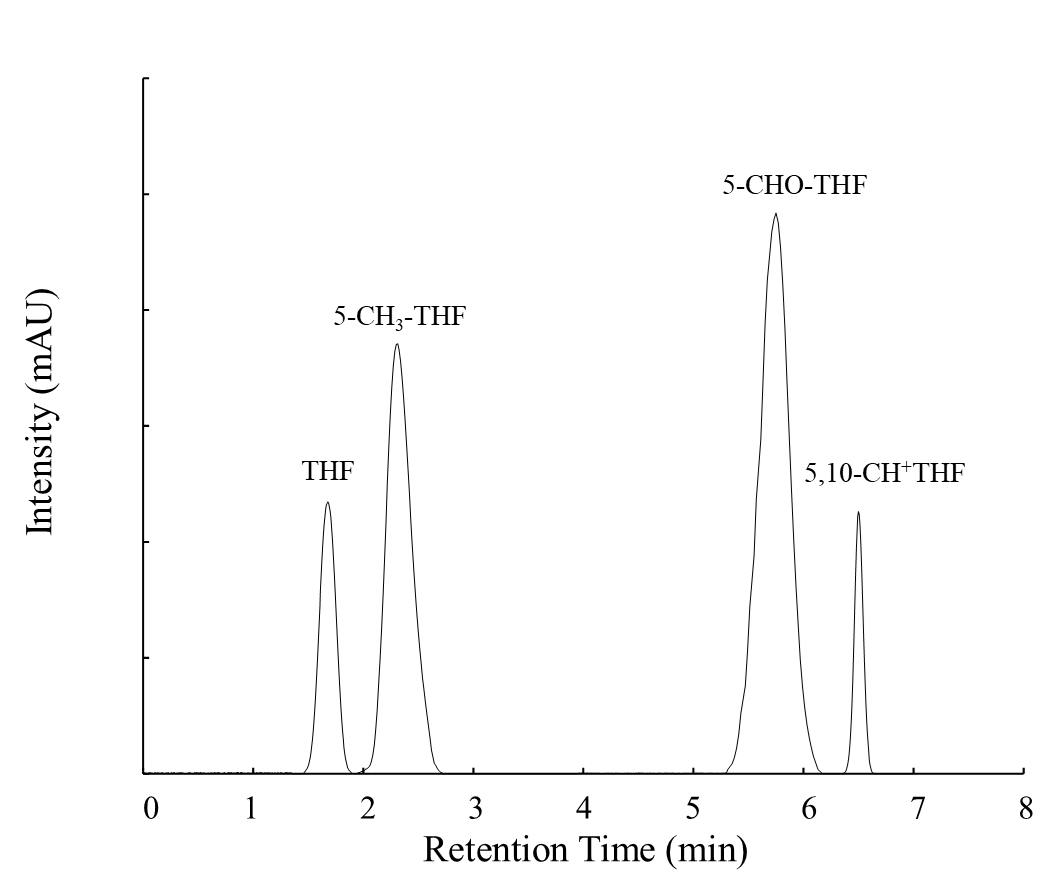
Supplementary Fig.2. High-performance liquid chromatography (HPLC) chromatograms of the standard mixture with UV detection.


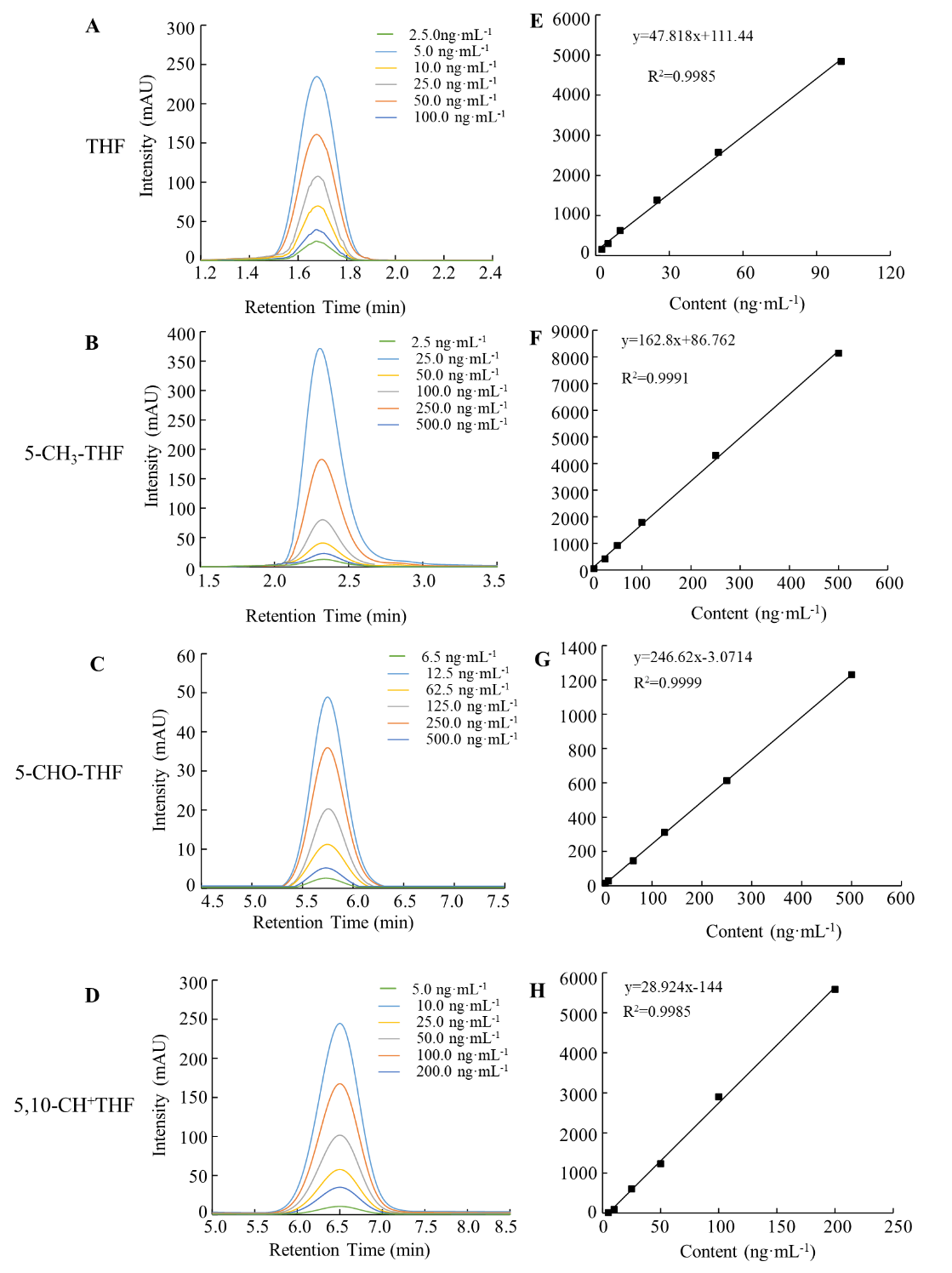


Supplementary Fig.3. HPLC chromatograms of folates with different concentrations and relationship between peak areas and folate contents. (**A, E**) THF, (**B, F**) 5-CH_3_-THF, (**C, G**) 5-CHO-THF, and (**D, H**) 5,10-CH^+^THF.


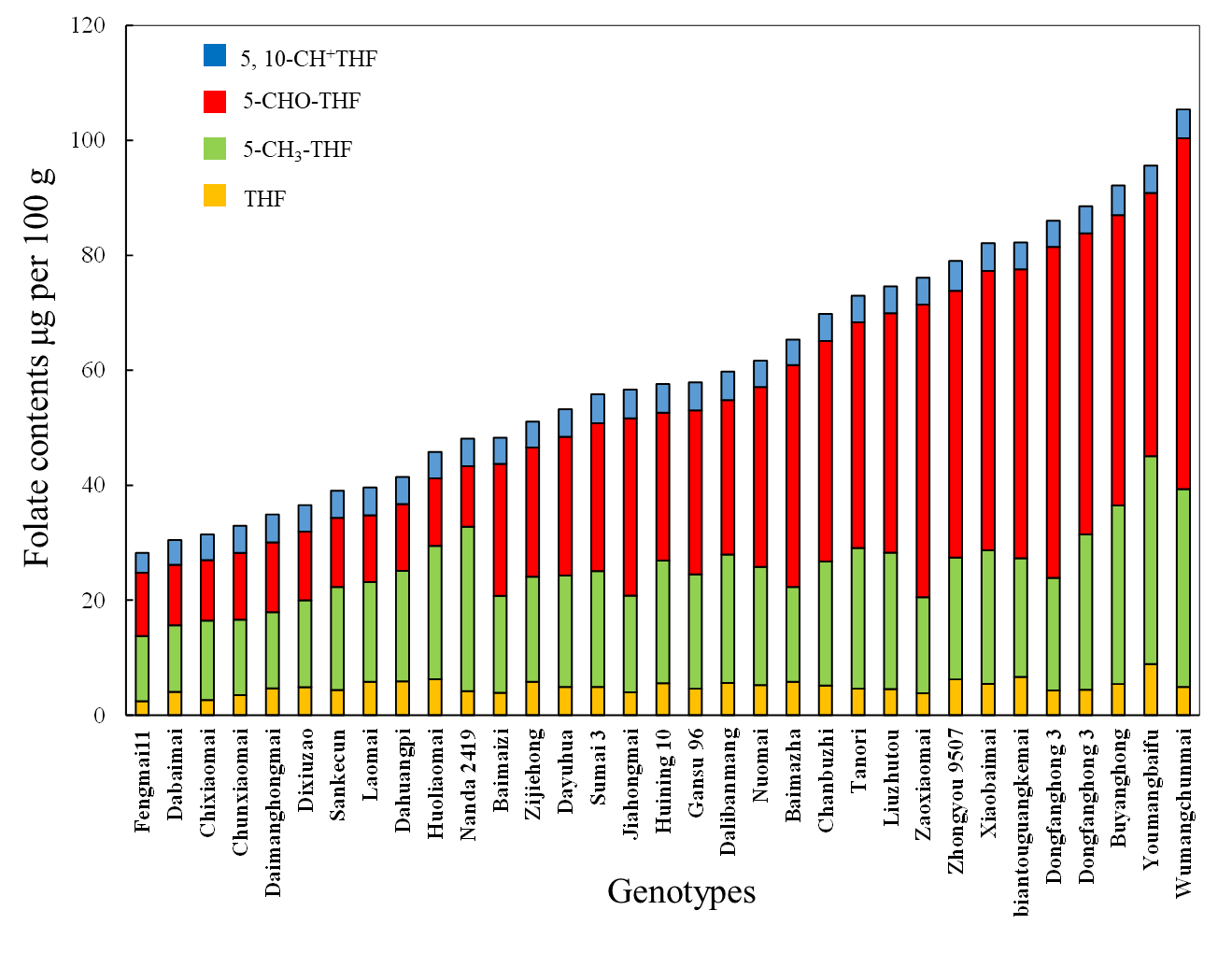


Supplementary Fig.4. Folate and its derivatives contents in MCC genotypes using the BLUP value in three environments. The distribution of folate derivatives of 5,10-CH^+^THF, 5-CHO-THF, 5-CH_3_-THF, and THF represented in different column types.
